# Preparing Multipartite Entangled Spin Qubits via Pauli Spin Blockade

**DOI:** 10.1038/s41598-020-60299-6

**Published:** 2020-02-26

**Authors:** Sinan Bugu, Fatih Ozaydin, Thierry Ferrus, Tetsuo Kodera

**Affiliations:** 1 Department of Electrical and Electronic Engineering, Tokyo Institute of Technology, 2-12-1 Ookayama, Meguro-ku, Tokyo, 152-8552 Japan; 2 Institute for International Strategy, Tokyo International University, 1-13-1 Matoba-kita, Kawagoe, Saitama, 350-1197 Japan; 3 Department of Information Technologies, Isik University, Sile, Istanbul, 34980 Turkey; 4 Hitachi Cambridge Laboratory, J. J. Thomson Avenue, CB3 0HE Cambridge, United Kingdom

**Keywords:** Quantum information, Information theory and computation, Quantum dots

## Abstract

Preparing large-scale multi-partite entangled states of quantum bits in each physical form such as photons, atoms or electrons for each specific application area is a fundamental issue in quantum science and technologies. Here, we propose a setup based on Pauli spin blockade (PSB) for the preparation of large-scale *W* states of electrons in a double quantum dot (DQD). Within the proposed scheme, two *W* states of *n* and *m* electrons respectively can be fused by allowing each *W* state to transfer a single electron to each quantum dot. The presence or absence of PSB then determines whether the two states have fused or not, leading to the creation of a *W* state of *n* + *m* − 2 electrons in the successful case. Contrary to previous works based on quantum dots or nitrogen-vacancy centers in diamond, our proposal does not require any photon assistance. Therefore the ‘complex’ integration and tuning of an optical cavity is not a necessary prerequisite. We also show how to improve the success rate in our setup. Because requirements are based on currently available technology and well-known sensing techniques, our scheme can directly contribute to the advances in quantum technologies and, in particular in solid state systems.

## Introduction

The preparation and manipulation of entangled systems is crucial for quantum technologies and more precisely, for quantum information. Despite the preparation of two-qubit entangled states, such as Einstein-Podolsky-Rosen (EPR) pairs, being well known, there is currently no available scheme for states made of multi-partite entangled states in general. The main reason lies in the fact that, in the multi-partite setting, states emerge in different classes, and transforming a state in a given class into a state in another class via stochastic local operations and classical communication (SLOCC) is, in general, not possible. Among basic classes, preparing an n-qubit Greenberger-Horne-Zeilinger (GHZ) state or a cluster (graph) state is relatively simple. That is, starting with one qubit in $\left(| 0\rangle +| 1\rangle \right)$/$\sqrt{2}$ state and *n* qubits each in  $| 0\rangle$ state, and applying *n* CNOT gates between these qubits (as qubit *i* the control and qubit *i* + 1 the target qubit for *i* = 1, 2, . . *n* − 1) one obtains a GHZ state in the form $\left(| {0}_{1}{0}_{2}{0}_{3}{..0}_{n}\rangle +| {1}_{1}{1}_{2}{1}_{3}{..1}_{n}\rangle \right)$/$\sqrt{2}$^1^. On the other hand, starting *n* qubits each in  $(| 0\rangle +| +\left|1\right\rangle )/\sqrt{2}$ state, one can obtain a cluster state in the desired form by applying a CZ gate between each pair of qubits accordingly^2^.

However, due to its more sophisticated structure, an intense effort has been devoted into developing methods for an efficient preparation of *W* states of *n*-qubits, which have form $\left(\left|{0}_{1}{0}_{2}{...0}_{n-1}{1}_{n}\right\rangle +\left|{0}_{1}{0}_{2}{...1}_{n-1}{0}_{n}\right\rangle \right)$ + $\left.\left|{0}_{1}{1}_{2}{...0}_{n-1}{0}_{n}\right\rangle +\left|{1}_{1}{0}_{2}{...0}_{n-1}{0}_{n}\right\rangle \right)$/$\sqrt{n}$. Instead of attempting at preparing the *W* state of multi-partite entangled states with a predetermined size from initially separable qubits, progressive approaches based on fusion or expansion of existing smaller size states have been widely recognized as a solution. First proposed and further improved for polarization-based encoded photons^3–12^, these approaches have been considered for electronic *W* states too, requiring atom-cavity interactions^13–18^, and also followed by different generation approaches, e.g., using quantum eraser and^19^, spin torque^20^, parity check gates^21^ and so on on^22,23^.

Solid state systems such as quantum dots and nitrogen-vacancy centers in diamond have also been considered for preparing *W* states of photonic qubits, as well as electronic qubits^24–26^. However, in these proposals, either photonic qubits are required for assisting the preparation process of electronic qubits, or conversely. Therefore, these proposals do require cavity-based systems to promote interactions between photons and electronic spins, rendering practical implementation far more challenging both technologically and experimentally. Despite the importance of such a scheme for quantum computation applications, there are currently no proposals for the preparation of *W* states that do not require a cavity. This is particularly true in solid-state systems and especially semiconductors whereas these are recognised as the most reliable and industry-compatible platforms for quantum information processing platforms. Within possible architectures, double quantum dots are one of the simplest systems in which spin qubits with reasonably long coherence time have been demonstrated^27,28^. In this work, considering spin of electrons in a double quantum dot (DQD) system, we propose a setup utilising the Pauli spin blockade (PSB) as a key element to fuse *W* states in a DQD system. PSB is a spin-based transport blockade due to Pauli exclusion principle and in a two electron regime DDQ system, if the electrons trapped in the dots have antiparallel spins, then interdot tunneling can exist, whilst the tunneling between the dots are blocked if the trapped electrons have parallel spins.

The PSB regime in DQDs is a promising candidate for probing fundamental physics. After being first observed in coupled quantum dots (QDs)^29^, this quantum effect was studied in a few-electron dots in combination with a sensor for reading the QD charge states^30–35^. PSB is now commonly used in several applications such as spin-to-charge conversion that has led to investigations into spin relaxation times^36,37^, electron spin coupling to nuclear spins^38^, as well as spin-orbit effects^39^.

The fusion approach for the *W* states was first introduced in ref. ^3^ when dealing with polarisation-based encoded photons. Similarly to photons, the proposal here is to access only a single electron in each *W* state to be fused in order to create a larger *W* state. We denote  ${\left|{W}_{n}\right\rangle }_{A}$ and  ${\left|{W}_{m}\right\rangle }_{B}$ the *W* states of two parties *A* and *B* and *n* and *m* their respective number of electrons, so we have:

1 $${| {W}_{n}\rangle }_{A}=\frac{1}{\sqrt{n}}\left({\left|{(n-1)}_{\downarrow }\right\rangle }_{a}{\left|{1}_{\uparrow }\right\rangle }_{1}+\sqrt{n-1}{\left|{W}_{n-1}\right\rangle }_{a}{\left|{1}_{\downarrow }\right\rangle }_{1}\right)$$

2 $${\left|{W}_{m}\right\rangle }_{B}=\frac{1}{\sqrt{m}}\left({\left|{(m-1)}_{\downarrow }\right\rangle }_{b}{\left|{1}_{\uparrow }\right\rangle }_{2}+\sqrt{m-1}{\left|{W}_{m-1}\right\rangle }_{b}{\left|{1}_{\downarrow }\right\rangle }_{2}\right)$$

**Figure 1:**
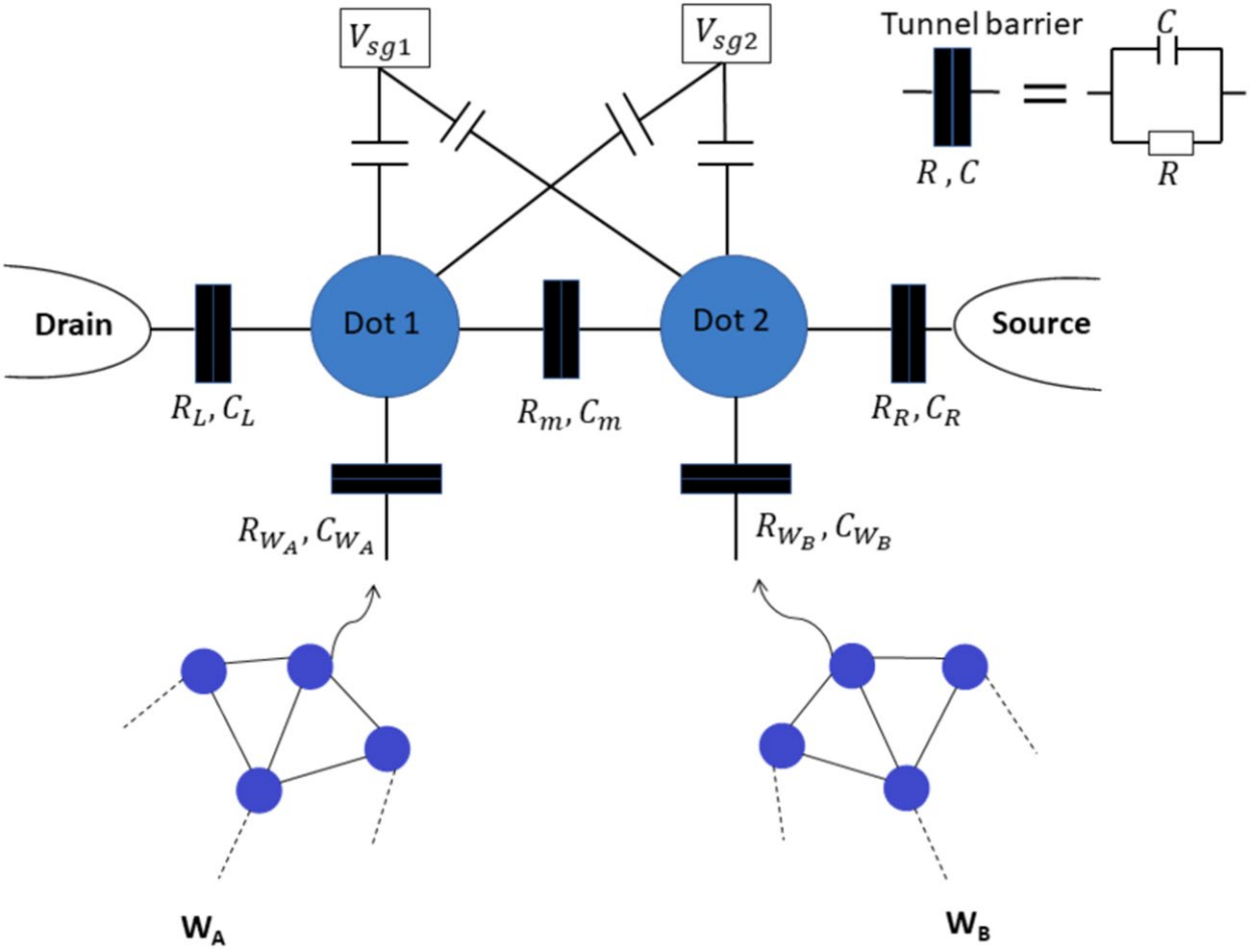
Network of driven (input) electrons and DQDs capacitively coupled with side gate 1 (sg1) and side gate 2 (sg2) which can tune the energy level of the dots. The two dots are tunnel-coupled (the subscripts R,m and L stand for right, mutual and left, respectively) with each other and with the source and drain. An attempt to fuse two *W* states, *W*_*A*_ and *W*_*B*_ via Pauli spin blockade (PSB) is as follows: One electron from each *W* state is sent to corresponding QD. Utilising charge sensing technique, observing no PSB implies a successful fusion as explained in the text.

Electrons labeled with 1(2) are sent to the fusion setup as illustrated in Fig. 1, and the electrons labeled with *a*(*b*) are kept at their site where $\left|\uparrow \right\rangle$ and $\left|\downarrow \right\rangle$ denote the spin up and spin down qubit states, respectively. In this notation, a three-electron W-state is written as ${\left|{W}_{3}\right\rangle }_{A}$ = $\frac{1}{\sqrt{3}}\left({\left|{2}_{\downarrow }\right\rangle }_{a}{\left|{1}_{\uparrow }\right\rangle }_{1}+\sqrt{2}{\left|{W}_{2}\right\rangle }_{a}{\left|{1}_{\downarrow }\right\rangle }_{1}\right)$ = $\frac{1}{\sqrt{3}}\left({\left|\downarrow \downarrow \uparrow \right\rangle }_{A}+{\left|\downarrow \uparrow \downarrow \right\rangle }_{A}+{\left|\uparrow \downarrow \downarrow \right\rangle }_{A}\right)$ with *W*_2_ corresponding to the EPR pair ${W}_{2}=\frac{1}{\sqrt{2}}\left(\left|\downarrow \uparrow \right\rangle +\left|\uparrow \downarrow \right\rangle \right)$.

## Results

In order to fuse two *W* states each with a single spin-up electron and create a larger *W* state, again with a single spin-up electron, one of the electrons either from *W*_*A*_ or *W*_*B*_ (subject to the fusion operation) should be found in a spin-up state, and the other in a spin-down state, i.e. the two electrons should have anti-parallel spins, without revealing which one is spin-up and which one is spin-down. The spin configuration of the two electrons is then revealed by charge sensing (Table 1). If electrons are found to have parallel spins, then two cases are possible:

**Table 1:** Four possible cases of fusion mechanism with corresponding probabilities. No Pauli spin blockade (PSB) implies a successful fusion of *W* states, whereas the presence of PSB implies either a recyclable case or a failure. Utilising charge sensor, absence of PSB implies a successful fusion.

Input	Probability	PSB	Result
*↓*, *↓*	$\frac{(n-1)(m-1)}{nm}$	Yes	Recycle
*↓*, *↑*	$\frac{(n-1)}{nm}$	No	Success
*↑*, *↓*	$\frac{(m-1)}{nm}$	No	Success
*↑*, *↑*	$\frac{1}{nm}$	Yes	Failure

(i) Both spins are spin-down and no fusion occurs. The *W* states are kept intact each with a single electron loss as
  3 $${\left|{W}_{n}\right\rangle }_{a}\otimes {\left|{W}_{m}\right\rangle }_{b}\to {\left|{W}_{n-1}\right\rangle }_{a}\otimes {\left|{W}_{m-1}\right\rangle }_{b}.$$
  However, one can attempt at fusing these two states again given that *n*, *m*≥2. Therefore this result is named ‘recyclable case’. There have been extensive analytical and numerical Monte Carlo simulations demonstrating that these recyclable states can reduce the resource cost in terms of the amount of entanglement spent to achieve a target size^3–6,40^.
(ii) Both spins are spin-up states. In this case, both *W* states have collapsed to a separable state with all the electrons in the spin-down state:
  4 $${\left|{W}_{n}\right\rangle }_{a}\otimes {\left|{W}_{m}\right\rangle }_{b}\to {\left|\downarrow \right\rangle }_{a}\otimes {\left|\downarrow \right\rangle }_{b}.$$
  Both *W* states are destroyed irrespectively of their sizes. There is no possibility of recycling the electrons and consequently, this is a ‘failure case’. We should stress that, as it is, this setup does not allow distinguishing the failure from the recycle case. Therefore, the recycle case can be regarded as a failure case at a cost of not utilising the recyclable states. Nevertheless, improvements in the scheme as described later will allow turning the failure case into a success case, removing the initial need for such a distinction.

However, if the electrons are found in opposite states, the fusion is achieved and the two *W* states are transformed into a larger single *W* state and

5 $${\left|{W}_{n}\right\rangle }_{a}\otimes {\left|{W}_{m}\right\rangle }_{b}\to \left|{W}_{n+m-2}\right\rangle .$$

The fusion scheme works as follow. Each *W* state provide each dot with a single electron by allowing tunneling via the side injection contacts A and B (Fig. 1). Once the electrons are added onto the QDs, the PSB is checked by the charge sensor and the spin states of the DQD are determined. In case of absence of PSB, either a current through the DQD is detected or a change in the reflective signal is observed, if one uses standard transport measurement or radio-frequency reflectometry^41^. This scheme works as long as the additional voltages applied to A and B that allow single electrons to tunnel through do not significantly disturb the PSB regime while avoiding electrons tunneling out of the dots via the source and drain reservoirs. In practice, such an electrostatic arrangement can be found by mapping the tunneling rates of the tunnel barriers and finding the appropriate tuning that allow the PSB measurement and the fusion to be done.

For the purpose of demonstration, the special case where *n* = *m* = 3 is discussed in details as this is the smallest *W* state number that our setup can fuse. In this case, and using Eqs. 2 and 3, the two states  ${\left|{W}_{3}\right\rangle }_{a}\otimes {\left|{W}_{3}\right\rangle }_{b}$ can be written as:

6 $$\frac{1}{\sqrt{3}}\left[\left({\left|\downarrow \uparrow \right\rangle }_{a}+{\left|\uparrow \downarrow \right\rangle }_{a}\right){\left|\downarrow \right\rangle }_{a}+{\left|\downarrow \downarrow \right\rangle }_{a}{\left|\uparrow \right\rangle }_{a}\right]\otimes \frac{1}{\sqrt{3}}\left[\left({\left|\downarrow \uparrow \right\rangle }_{b}+{\left|\uparrow \downarrow \right\rangle }_{b}\right){\left|\downarrow \right\rangle }_{b}+{\left|\downarrow \downarrow \right\rangle }_{b}{\left|\uparrow \right\rangle }_{b}\right].$$

The third electron will be used as a fusion enabler. If PSB is detected then both electrons are spin-down states and we are in the ‘recycle case’. This leaves each *W* states with one electron missing and so:

7 $$\frac{1}{\sqrt{2}}\left({\left|\downarrow \uparrow \right\rangle }_{a}+{\left|\uparrow \downarrow \right\rangle }_{a}\right)\otimes \frac{1}{\sqrt{2}}\left({\left|\downarrow \uparrow \right\rangle }_{b}+{\left|\uparrow \downarrow \right\rangle }_{b}\right).$$

If both electrons are found in spin-up states, the process failed to realise the fusion. Both *W* states are in a separable system of spin-down states, again each with one missing electron. Consequently, we have:

$${\left|\downarrow \downarrow \right\rangle }_{a}\otimes {\left|\downarrow \downarrow \right\rangle }_{b}.$$

However, the absence of PSB implies that the spins are anti-parallel. If the first one is spin-down and the second is spin-up, then the result is

8 $$\frac{1}{\sqrt{2}}\left(\left|\downarrow \uparrow \right\rangle +\left|\uparrow \downarrow \right\rangle \right)\otimes \left|\downarrow \downarrow \right\rangle =\frac{1}{\sqrt{2}}\left(\left|\downarrow \uparrow \downarrow \downarrow \right\rangle +\left|\uparrow \downarrow \downarrow \downarrow \right\rangle \right),$$

Alternatively, we end up with:

9 $$\left|\downarrow \downarrow \right\rangle \otimes \frac{1}{\sqrt{2}}\left(\left|\downarrow \uparrow \right\rangle +\left|\uparrow \downarrow \right\rangle \right)=\frac{1}{\sqrt{2}}\left(\left|\downarrow \downarrow \downarrow \uparrow \right\rangle +\left|\downarrow \downarrow \uparrow \downarrow \right\rangle \right).$$

As the absence of PSB leaves the system in the superposition of these two states, the result is a genuine *W* state of 4 electrons

10 $$\frac{1}{\sqrt{4}}\left(\left|\downarrow \uparrow \downarrow \downarrow \right\rangle +\left|\uparrow \downarrow \downarrow \downarrow \right\rangle +\left|\downarrow \downarrow \downarrow \uparrow \right\rangle +\left|\downarrow \downarrow \uparrow \downarrow \right\rangle \right),$$

implying a successful fusion operation.

Provided that the PSB sensing is performed much faster than the coherence time and that the remaining electrons of the *W* states are kept undisturbed in their quantum memories, the proposed scheme can evaluate the success of the state preparation as well as realise the fusion process itself (Table 1). Despite the drawback of loosing two qubits and an inherently probabilistic success, this is an advantage over deterministic preparation approaches, where multi-qubit controlled gates are required^9,10^. Both our proposal for electron spins (Fig. 1) and Özdemir’ scheme^3^ for photons can fuse *W* states containing a minimum of 3-qubits each, the preparation of 2-qubit *W* states requiring a dedicated scheme.

**Figure 2:**
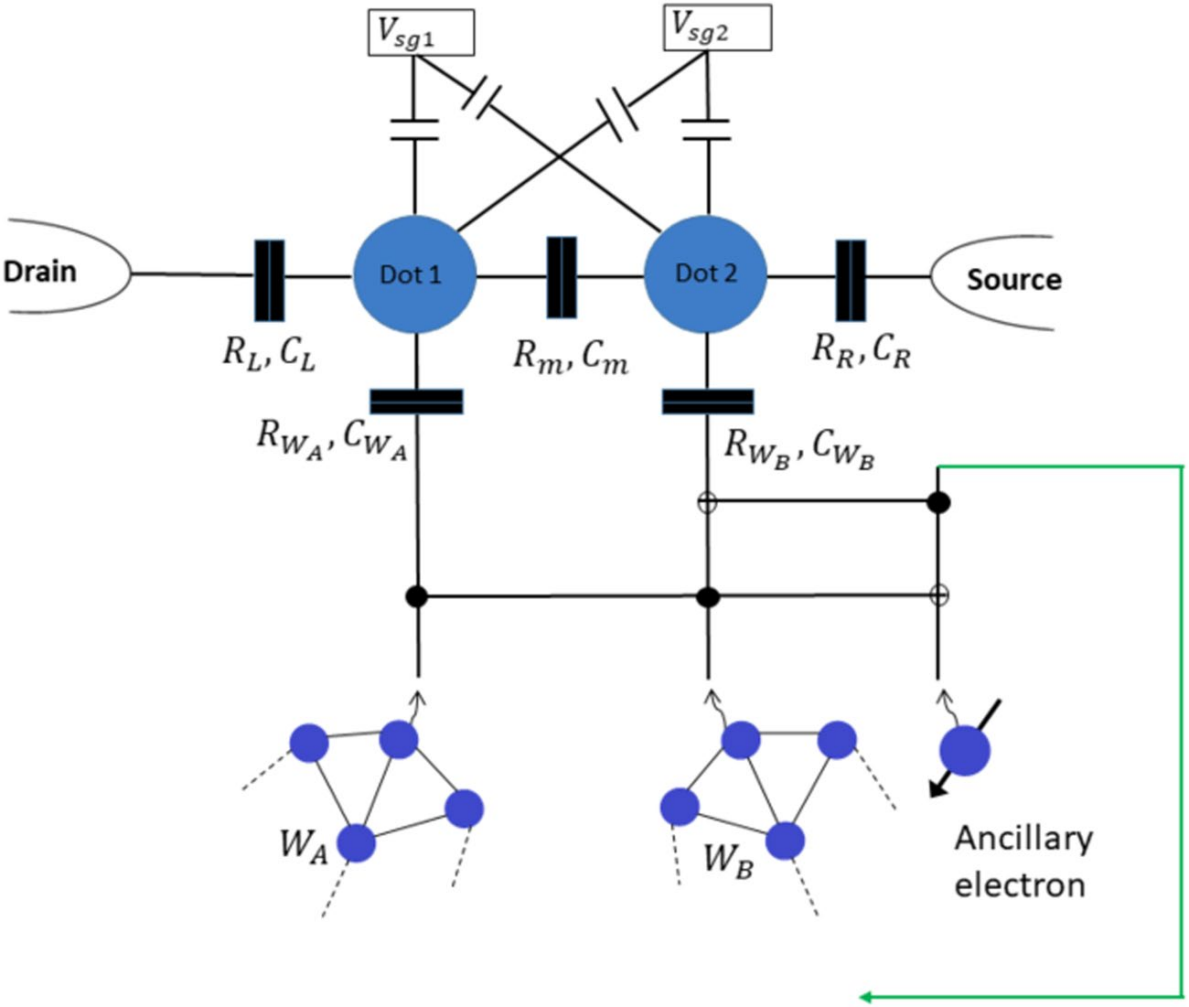
Proposed enhanced mechanism for transforming the failure into a success case. One electron from each *W* state and an ancillary spin-down electron is sent to the Toffoli+CNOT gate before reaching the fusion setup. Again, the absence of PSB implies a successful fusion.

While the recycle case only decreases the size of the initial states by one qubit, the failure case is catastrophic for the large-scale *W* states as both *W* states are destroyed. In order to overcome this problem, an enhanced fusion proposal was put forward in ref. ^4^ to integrate a Fredkin gate and to employ an ancillary photon. It was later demonstrated that when using a Fredkin gate - that is realised by one Toffoli gate and two CNOT gates, only one Toffoli gate and one CNOT gate are indeed sufficient to realise the enhanced fusion operation^8^, due to limited number of input states. A similar approach can be taken here. A Toffoli gate followed by a CNOT gate is placed before the DQD device (Fig. 2). Qubits coming from the *W* states act as control qubits while an ancillary electron in spin-down state acts as the target qubit. This allows performing the operations

11 $$\begin{array}{rcl}(\mathrm{Recycle})\ \ \left|\downarrow \right\rangle \left|\downarrow \right\rangle \left|\downarrow \right\rangle  & \to  & \left|\downarrow \right\rangle \left|\downarrow \right\rangle \left|\downarrow \right\rangle \ \ (\mathrm{Recycle}),\\ (\mathrm{Success})\ \ \left|\downarrow \right\rangle \left|\uparrow \right\rangle \left|\downarrow \right\rangle  & \to  & \left|\downarrow \right\rangle \left|\uparrow \right\rangle \left|\downarrow \right\rangle \ \ (\mathrm{Success})\\ (\mathrm{Success})\ \ \left|\uparrow \right\rangle \left|\downarrow \right\rangle \left|\downarrow \right\rangle  & \to  & \left|\uparrow \right\rangle \left|\downarrow \right\rangle \left|\downarrow \right\rangle \ \ (\mathrm{Success})\\ (\mathrm{Fail})\ \ \left|\uparrow \right\rangle \left|\uparrow \right\rangle \left|\downarrow \right\rangle  & \to  & \left|\uparrow \right\rangle \left|\downarrow \right\rangle \left|\uparrow \right\rangle \ \ (\mathrm{Success}),\end{array}$$

and converts the ‘failure case’ into a ‘successful one’. We should stress that, in the latter, the third qubit is returned back to the fused *W* state, thus increasing the final size by one qubit. The minimum size for the *W* states required to implement this scheme is also reduced from three to two qubits, allowing EPR pairs to be fused.

In our improved setup, the enhancement is achieved as long as the gate operations are performed before electrons enter the DQD device and so, it is not a requirement to build these gates at the DQD level, which simplify drastically the device design and voltage pulsing scheme. Zajac *et al*. have recently demonstrated CNOT gate operations in a DQD^42^. Since Toffoli gates can be realised in terms of CNOT gates, such a setup for electrons can be realised in DQDs with current technology. CNOT and Toffoli gates can also be realised for electrons in hybrid systems^43,44^, implying there may be several alternative implementation techniques for our enhanced setup, which could possible utilise the Hadamard gate for spin qubits in QDs performing the operation^27^  $\left|\uparrow \right\rangle \to \left(\left|\uparrow \right\rangle +\left|\downarrow \right\rangle \right)$/$\sqrt{2}$ and  $\left|\downarrow \right\rangle \to \left(\left|\uparrow \right\rangle -\left|\downarrow \right\rangle \right)$/$\sqrt{2}$, as already realized^45^.

However, one critical issue is noise, in particular charge noise and spin orbit coupling, which limits the coherence time of the electron spins in the DQD. With progress in fabrication techniques like etching and surface reconstruction, improvement in material quality^46^ and advancement in pulsing schemes (dynamical decoupling like the Carr-Purcell-Meiboom-Gill pulse protocol^47,48^), the coherence time have been significantly extended to few tens of *μ*s for QDs^49^ or even tens of ms for spins in DQDs^28^. The state preparation in our setup consists of two basic steps: the injection of electrons from the *W* states into the DQD and the PSB sensing. The former can be performed in a few hundreds of picoseconds^50^. Depending on the method employed, the latter can be realised in a few ns if one uses radiofrequency reflectometry^51,52^ to few hundreds of ns for other methods^53^. In all cases, the spin is conserved during the tunnelling events. Indeed, the non spin conservation probability is proportional to the difference in the g-factors across the barrier. However, the uniformity of the material and the symmetry of the quantum dots render this probability negligible in practice^54^. This makes the experimental realisation of fused states possible with the current state-of-art technologies and techniques. Experimental non-idealities is obviously affecting the success of the state preparation. Assuming that the probability for an electron spin (taken from a *W* state) to be flipped during the overall process is *p*, the possible output probabilities are: (i) *p*(1 − *p*) for the recycle case, (ii) (1−*p*)^2^ + *p*^2^ in case of success and, (iii) *p*(1 − *p*) for a failure case. Therefore, the resultant state is not a pure $\left|{W}_{n+m-2}\right\rangle$ state anymore but a mixture of these possible states, i.e.

12 $$\begin{array}{rl}\left|{W}_{n}\right\rangle \left\langle {W}_{n}\right|\otimes \left|{W}_{m}\right\rangle \left\langle {W}_{m}\right| & \to \ p(1-p)\left(\left|{W}_{n-1}\right\rangle \left\langle {W}_{n-1}\right|\otimes \left|{W}_{m-1}\right\rangle \left\langle {W}_{m-1}\right|\right)\\  & +\ [{(1-p)}^{2}+{p}^{2}]\left(\left|{W}_{n+m-2}\right\rangle \left\langle {W}_{n+m-2}\right|\right)\\  & +\ p(1-p){\left(\left|\downarrow \right\rangle \left\langle \downarrow \right|\right)}^{\otimes n+m-2},\end{array}$$

This yields to a fidelity of (1−*p*)^2^ + *p*^2^ for the expected $\left|{W}_{n+m-2}\right\rangle$ state. Because of this, there will be occasions where the result may be interpreted with some probability as a recycle or a failure and so, the successfully prepared *W*_*n*+*m*−2_ state may be wrongly discarded in these cases. This increase in the resource cost *R* as been discussed elsewhere^3^ and, in the ideal case, *R*[*W*_*n*+*m*−2_] = (*R*[*W*_*n*_] + *R*[*W*_*m*_])∕*P*_*s*_(*n*, *m*), where *P*_*s*_(*n*, *m*) is the probability of successfully fusing two *W* states of *n* and *m* qubits, and *R*[*W*_*n*_] the resource cost of a *W* state of *n* qubits.

In a noisy setup, a successful case will be detected with probability (1−*p*)^2^ + *p*^2^ and so, the new resource cost becomes

13 $${R}^{{\prime} }[{W}_{n+m-2}]=\frac{{R}^{{\prime} }[{W}_{n}]+{R}^{{\prime} }[{W}_{m}]}{{P}_{s}(n,m)[{(1-p)}^{2}+{p}^{2}]}.$$

The expected probability of spin flips during the fusion process is relatively small in practice, because in the absence of an external magnetic field, the probability of spin flip is linearly dependent on the electron concentration and inversely proportional the lifetime of spins. In the low electron regime, the latter become preponderant and depends on the spin-orbit coupling (SOC) strength. In the absence of interface and even surface, the SOC is generally negligible in silicon but somehow more important in III-V material due to the intrinsic presence of non-zero nuclear spin moments. However, the previous derivations of the fidelity and the resource cost do apply to any *W*-state fusion process for any physical qubits in a relevant fusion mechanism.

## Discussion

We should mention that the previous discussion concerns the possibility of implementing the fusion process with currently available technology only, and not the way of storing a multipartite entangled state in a quantum memory which is itself a separate problem. For each physical qubit (atomic, photonic or electronic), the basic assumption in preparing a large-scale multipartite entangled state is either having a suitable quantum memory, or preparing the setup in a scalable way so that no memory is required and the prepared entangled state is immediately used in the algorithm within the coherence time of the state. On the other hand, as photonic qubits are optimal for communication, solid state qubits are optimal for storage. In particular, electrons in quantum dots are promising for their long coherence times, suggesting that preparing a large-scale multipartite entangled state of electronic qubits would be much practical than photonic qubits, for example.

We have proposed a simple setup for preparing large-scale *W* states of electron spins, based on Pauli spin blockade in DQD devices that could be implemented with the currently available technologies. Unlike previous proposals, our scheme does not require photon assistance and therefore cavity-based systems. We also described how to enhance the basic fusion setup using logic gates and an ancillary electron spin. We believe the fusion scheme we proposed in this study has the potential to open up a number of new quantum dots-based multipartite entanglement creation and manipulation mechanisms.

## Methods

In general, DQD devices have both dots coupled to each other and to the reservoirs by tunnel barriers but are only capacitively coupled to gate electrodes. The latter aims at controlling energy levels of individual dot as well as the tunneling rates between the two dots. The charge configuration of the DQD is generally depicted by the pair (*n*, *m*) that corresponds to the number of electrons on the left and right dot, respectively. Here, the DQD is tuned such that the charge transitions involve the transport cycle (0, 1) → (1, 1) → (0, 2) → (0, 1) in the PSB regime. Simple model of two electrons in a DQD can be written in the basis of three charge configurations $\left|20\right\rangle$ (both electrons left), $\left|11\right\rangle$ (electrons split) and $\left|02\right\rangle$ (both electrons right):

14 $$H=\left(\begin{array}{ccc}{H}_{20} & T & 0\\ T & {H}_{11} & T\\ 0 & T & {H}_{02}\end{array}\right)$$

**Figure 3:**
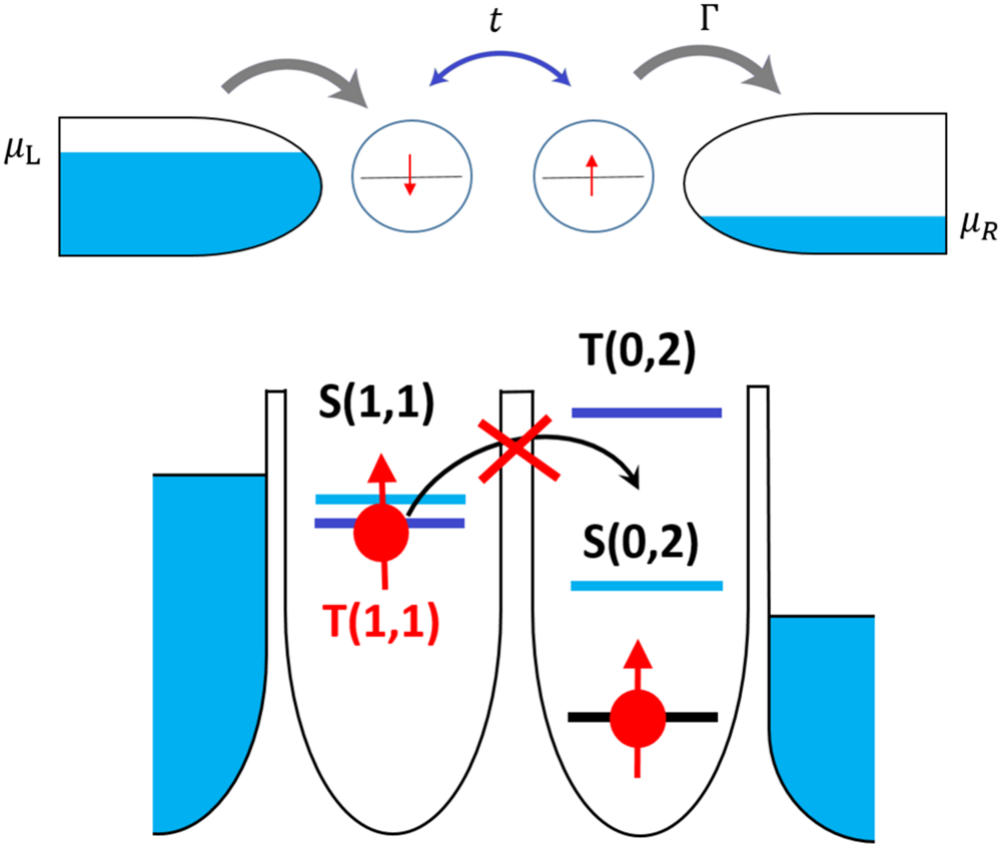
DQD in the Pauli spin blockade regime. (above) Quantum dots are coupled to source and drain. Because of the energy difference tuned by side gates, electrons can only run from the left to the right lead. *t* is inter-dot tunnel coupling and *Γ* is tunneling rate. (below) Energy diagram of spin-conserving inter-dot coupling. Since the only accessible (0,2) state is a spin singlet, all (1,1) states are not coupled to the (0,2) state and hence the current is blocked.

Transport between QDs is determined by the relative positions of their electrochemical potentials *μ*_*l*_(*n*, *m*) ≡ *E*(*n*, *m*) − *E*(*n* − 1, *m*) and *μ*_*r*_(*n*, *m*) ≡ *E*(*n*, *m*) − *E*(*n*, *m* − 1), where *E* denotes the total energy of the system and *n* and *m* denote number of electrons in left and right quantum dot, respectively^55^. In the absence of an external magnetic field, the ground state is a singlet state formed by a pair of electrons with anti-parallel spins and localised on each quantum dot. In this case, inter-dot tunneling is allowed and current flows through the device (Fig. 3 (above)). On the contrary, if electrons have parallel spins (triplet state), the tunneling between the dots is blocked due to Pauli exclusion principle and no inter-dot tunneling occurs (Fig. 3 (below)). However, in non-ideal conditions, electron tunneling between dots in PSB regime can also be observed due to leakage processes which induce spin-flips and singlet to triplet transitions. These cases fall mostly under two categories depending on their origin:

(i) Spin-orbit coupling (SOC): the electron spins interact with the magnetic field created by the tunneling of electrons between the two dots and the surrounded electric field induced either naturally by the crystal lattice (Rashba or Dresselhaus) or by an external voltage bias. In this situation, the spin degeneracy is lifted and the fluctuations in the electron-phonon interaction may compensate the Zeeman energy, leading to spin relaxation^56^.
(ii) Hyperfine interaction: Interaction of electron spins with the surrounding nuclear spins can induce a similar process, mixing the spin states *T*(1, 1) with *S*(1, 1) and resulting in *T*(1, 1) → *S*(1, 1) → *S*(2, 0) transition leakage current^57–59^. Despite this mechanism, it is possible to infer whether electrons in the DQD had anti-parallel or parallel spins initially. In order to avoid misinterpretation of the state preparation in the results, this effect is included in our model.

## References

[1] Nielsen, M. A. & Chuang, I. L. *Quantum Computation and Quantum Information: 10th Anniversary Edition*, 10th edn. (Cambridge University Press, USA, 2011).

[2] Raussendorf R, Briegel HJ (2001). A one-way quantum computer. Phys. Rev. Lett..

[3] Ozdemir SK (2011). An optical fusion gate for W-states. New J. Phys..

[4] Bugu S, Yesilyurt C, Ozaydin F (2013). Enhancing the W-state quantum-network-fusion process with a single Fredkin gate. Phys. Rev. A.

[5] Yesilyurt C, Bugu S, Ozaydin F (2013). An optical gate for simultaneous fusion of four photonic W or Bell states. Quant. Info. Proc..

[6] Ozaydin F (2014). Fusing multiple W states simultaneously with a Fredkin gate. Phys Rev. A.

[7] Li K (2016). Generating multi-photon W-like states for perfect quantum teleportation and superdense coding. Quant. Inf. Process..

[8] Diker F, Ozaydin F, Arik M (2015). Enhancing the W state fusion process with a Toffoli gate and a CNOT gate via one-way quantum computation and linear optics. Acta Phys. Pol. A.

[9] Yesilyurt C, Bugu S, Diker F, Altintas AA, Ozaydin F (2015). An optical setup for deterministic creation of four partite W state. Acta Phys. Pol. A.

[10] Yesilyurt C (2016). Deterministic local doubling of W states. J. Opt. Soc. Am. B.

[11] Eibl M, Kiesel N, Bourennane M, Kurtsiefer C, Weinfurter H (2004). Experimental realization of a three-qubit entangled W state. Phys. Rev. Lett..

[12] Heo, J., Hong, C., Choi, S.-G. & Hong, J.-P. Scheme for generation of three-photon entangled W state assisted by cross-Kerr nonlinearity and quantum dot. *Sci. Rep*. **9**, 10.1038/s41598-019-46231-7 (2019). 10.1038/s41598-019-46231-7 PMC6626062 31300664

[13] YANG MING, YI YOU-MING, CAO ZHUO-LIANG (2004). SCHEME FOR PREPARATION OF W STATE VIA CAVITY QED. International Journal of Quantum Information.

[14] Deng ZJ, Feng M, Gao KL (2006). Simple scheme for generating an n-qubit W state in cavity QED. Phys. Rev. A.

[15] Zang X-P, Yang M, Ozaydin F, Song W, Cao Z-L (2015). Generating multi-atom entangled W states via light-matter interface based fusion mechanism. Sci. Rep..

[16] Zang X-P, Yang M, Ozaydin F, Song W, Cao Z-L (2015). Deterministic generation of large scale atomic W states. Opt. Exp(11).

[17] Zang XP, Yang M, Wang XC, Song W, Cao ZL (2015). Fusion of W states in a cavity quantum electrodynamic system. Can. J. Phys..

[18] Sheng Y-B, Zhou L (2013). Efficient W-state entanglement concentration using quantum-dot and optical microcavities. J. Opt. Soc. Am. B.

[19] Kim, Y.-S., Cho, Y.-W., Lim, H.-T. & Han, S.-W. Efficient generation of multipartite W state via quantum eraser. *arXiv e-prints* arXiv:1911.12971, 1911.12971 (2019).

[20] Sharma, A. & Tulapurkar, A. A. Generation of n-qubit W states using Spin Torque. *arXiv e-prints* arXiv:1912.03171, 1912.03171 (2019).

[21] Sheng YuBo, Pan Jun, Guo Rui, Zhou Lan, Wang Lei (2015). Efficient N-particle W state concentration with different parity check gates. Science China Physics, Mechanics & Astronomy.

[22] Sheng Y-B, Zhou L, Zhao S-M (2012). Efficient two-step entanglement concentration for arbitrary W states. Phys. Rev. A.

[23] Wu, N.-N. & Jiang, M. A highly efficient scheme for joint remote preparation of multi-qubit W state with minimum quantum resource. *Quant. Info. Proc*. **17**, 10.1007/s11128-018-2098-0 (2018).

[24] Han X (2015). Effective W-state fusion strategies for electronic and photonic qubits via the quantum-dot-microcavity coupled system. Sci. Rep..

[25] Li N, Yang J, Ye L (2015). Realizing an efficient fusion gate for W states with Cross-Kerr nonlinearities and QD-cavity coupled system. Quant. Inf. Process.

[26] Han X, Guo Q, Zhu A-D, Zhang S, Wang H-F (2017). Effective W-state fusion strategies in nitrogen-vacancy centers via coupling to microtoroidal resonators. Opt. Exp..

[27] Loss D, DiVincenzo DP (1998). Quantum computation with quantum dots. Phys. Rev. A.

[28] Veldhorst M (2014). An addressable quantum dot qubit with fault-tolerant control-fidelity. Nature Nanotechnology.

[29] Ono K, Austing DG, Tokura Y, Tarucha S (2002). Current rectification by pauli exclusion in a weakly coupled double quantum dot system. Science.

[30] Elzerman JM (2003). Few-electron quantum dot circuit with integrated charge read out. Phys. Rev. B.

[31] Johnson AC, Petta JR, Marcus CM, Hanson MP, Gossard AC (2005). Singlet-triplet spin blockade and charge sensing in a few-electron double quantum dot. Phys. Rev. B.

[32] Johnson AC (2005). Triplet-singlet spin relaxation via nuclei in a double quantum dot. Nature.

[33] Zhang LX, Leburton JP, Hanson R, Kouwenhoven LP (2004). Engineering the quantum point contact response to single-electron charging in a few-electron quantum-dot circuit. Appl. Phys. Lett..

[34] Yang CH (2013). Spin-valley lifetimes in a silicon quantum dot with tunable valley splitting. Nat. Commun..

[35] Morello A (2010). Single-shot readout of an electron spin in silicon. Nature.

[36] Petta JR (2005). Pulsed-gate measurements of the singlet-triplet relaxation time in a two-electron double quantum dot. Phys. Rev. B.

[37] Hu Y, Kuemmeth F, Lieber CM, Marcus CM (2012). Hole spin relaxation in ge-si core-shell nanowire qubits. Nature Nanotechnology.

[38] Ono K, Tarucha S (2004). Nuclear-spin-induced oscillatory current in spin-blockaded quantum dots. Phys. Rev. Lett..

[39] Nadj-Perge S (2010). Disentangling the effects of spin-orbit and hyperfine interactions on spin blockade. Phys. Rev. B.

[40] Li K, Kong F-Z, Yang M, Yang Q, Cao Z-L (2016). Qubit-loss-free fusion of W states. Phys. Rev. A.

[41] Schoelkopf RJ, Wahlgren P, Kozhevnikov AA, Delsing P, Prober DE (2008). The radio-frequency single-electron transistor (rf-set): A fast and ultrasensitive electrometer. Science.

[42] Zajac DM (2018). Resonantly driven CNOT gate for electron spins. Science.

[43] Wei HR, Deng FG (2014). Universal quantum gates on electron-spin qubits with quantum dots inside single-side optical microcavities. Opt. Express.

[44] Luo MX, Ma SY, Chen XB, Wang X (2015). Hybrid Toffoli gate on photons and quantum spins. Sci. Rep..

[45] Kawakami Erika, Jullien Thibaut, Scarlino Pasquale, Ward Daniel R., Savage Donald E., Lagally Max G., Dobrovitski Viatcheslav V., Friesen Mark, Coppersmith Susan N., Eriksson Mark A., Vandersypen Lieven M. K. (2016). Gate fidelity and coherence of an electron spin in an Si/SiGe quantum dot with micromagnet. Proceedings of the National Academy of Sciences.

[46] Abrosimov NV (2017). A new generation of 99.999% enriched ^28^Si single crystals for the determination of Avogadro’s constant. Metrologia.

[47] Carr HY, Purcell EM (1954). Effects of diffusion on free precession in nuclear magnetic resonance experiments. Phys. Rev..

[48] Meiboom S, Gill D (1958). Modified spin-echo method for measuring nuclear relaxation times. Review of Scientific Instruments.

[49] Yoneda J (2018). A quantum-dot spin qubit with coherence limited by charge noise and fidelity higher than 99.9. Nature Nanotech.

[50] Talalaev VG (2008). Transient carrier transfer in tunnel injection structures. Applied Physics Letters.

[51] Schoelkopf RJ, Wahlgren P, Kozhevnikov AA, Delsing P, Prober DE (1998). The radio-frequency single-electron transistor (RF-SET): A fast and ultrasensitive electrometer. Science.

[52] Ahmed I (2018). Radio-frequency capacitive gate-based sensing. Phys. Rev. Applied.

[53] Vink IT, Nooitgedagt T, Schouten RN, Vandersypen LMK (2007). Cryogenic amplifier for fast real-time detection of single-electron tunneling. Appl. Phys. Lett..

[54] Roy D (2013). Spin noise spectroscopy of quantum dot molecules. Phys. Rev. B.

[55] van der Wiel WG (2002). Electron transport through double quantum dots. Rev. Mod. Phys..

[56] Wang J-Y (2018). Anisotropic pauli spin-blockade effect and spin-orbit interaction field in an InAs nanowire double quantum dot. Nano Lett..

[57] Koppens FHL (2011). Control and detection of singlet-triplet mixing in a random nuclear field. Phys. Rev. B.

[58] Pfund A, Shorubalko I, Ensslin K, Leturcq R (2007). Spin-state mixing in InAs double quantum dots. Phys. Rev. B.

[59] Nadj-Perge S (2012). Spectroscopy of spin-orbit quantum bits in indium antimonide nanowires. Phys. Rev. Lett..

